# Favourable 16-year outcome of an acetabular reinforcement ring with suboptimal host-bone coverage: the role of biological and mechanical stabilisation

**DOI:** 10.1093/jscr/rjag461

**Published:** 2026-06-13

**Authors:** João Castro Mendes, João Leite Moreira, João Seixas, Catarina Corte-Real, Daniela Isidoro, Pedro Farinha, José Rodrigues, Fernando Judas

**Affiliations:** Orthopaedic Department, ULS Coimbra, Praceta Professor Mota Pinto, Celas, 3004-561 Coimbra, Portugal; Orthopaedic Department, ULS Coimbra, Praceta Professor Mota Pinto, Celas, 3004-561 Coimbra, Portugal; Orthopaedic Department, ULS Coimbra, Praceta Professor Mota Pinto, Celas, 3004-561 Coimbra, Portugal; Orthopaedic Department, ULS Coimbra, Praceta Professor Mota Pinto, Celas, 3004-561 Coimbra, Portugal; Orthopaedic Department, ULS Coimbra, Praceta Professor Mota Pinto, Celas, 3004-561 Coimbra, Portugal; Orthopaedic Department, ULS Coimbra, Praceta Professor Mota Pinto, Celas, 3004-561 Coimbra, Portugal; Orthopaedic Department, ULS Coimbra, Praceta Professor Mota Pinto, Celas, 3004-561 Coimbra, Portugal; Orthopaedic Department, ULS Coimbra, Praceta Professor Mota Pinto, Celas, 3004-561 Coimbra, Portugal

**Keywords:** developmental dysplasia of the hip, acetabular dysplasia, total hip arthroplasty, acetabular reinforcement ring, cone prosthesis stem

## Abstract

Total hip arthroplasty for end-stage dysplastic osteoarthritis remains technically demanding because of altered acetabular and femoral anatomy. In 2010, a 59-year-old man with Crowe I dysplasia and Tönnis 3 osteoarthritis underwent total hip arthroplasty using a roof reinforcement ring and cemented polyethylene cup. Because of suboptimal acetabular reaming and insufficient medialisation, a substantial superolateral segment of the ring remained uncovered. This defect was managed with impacted morselized femoral head autograft, and femoral reconstruction was performed with a cementless cone stem. Despite concerns regarding primary mechanical stability, 16-year follow-up showed an asymptomatic and fully functional hip. Radiographs demonstrated graft incorporation, secondary biological stabilisation of the ring, and no evidence of implant instability or loosening. Moderate acetabular wear and mature heterotopic ossification were present. This case highlights that incorporation of morselised femoral head autograft may compensate for insufficient initial host-bone coverage and support durable long-term survival in dysplastic acetabular reconstruction.

## Introduction

Total hip arthroplasty (THA) is a common and highly effective procedure. Modern techniques provide substantial pain relief and restore hip mobility for prolonged periods in patients with debilitating hip dysfunction [1, 2]. However, all hip replacements will ultimately fail if they remain in situ long enough, as these implants have a finite lifespan. Nevertheless, THA remains the procedure of choice for most patients with symptomatic end-stage osteoarthritis secondary to hip dysplasia [3].

Developmental dysplasia of the hip (DDH) encompasses a spectrum of abnormalities involving acetabular and proximal femoral dysplasia, hip instability, subluxation and dislocation. The dysplastic acetabulum is typically shallow, narrow and lateralized, with increased anteversion and deficiency of the anterior and superior walls. Femoral deformities commonly include excessive neck version, posterior displacement of the greater trochanter, a valgus neck-shaft angle, hypoplasia of the intramedullary canal, rotational metaphyseal-diaphyseal mismatch and abductor muscle contracture. DDH is a well-recognized cause of secondary hip osteoarthritis and frequently results in inadequate coverage of the degenerative femoral head [4, 5]. Total hip arthroplasty for end-stage dysplastic osteoarthritis remains technically demanding, requiring thorough understanding of the anatomical abnormalities and proficiency in complex reconstructive techniques. Careful preoperative planning, with consideration of the available reconstruction strategies, is essential to achieve a successful outcome [5].

In this report we present the long-term outcomes of a THA performed to treat osteoarthritis secondary to DDH. A roof reinforcement ring with a cemented polyethylene cup and a cone prosthesis stem were implanted. Due to to suboptimal acetabular reaming and insufficient acetabular medialisation, the superolateral portion of the ring was uncovered by host bone. Early mechanical failure of the implant was initially anticipated due to these technical limitations. However, an unexpectedly favourable result was observed at 5-year follow-up and previously published [6] demonstrating the mechanical stability of the THA.

The present report provides an updated 16-year follow-up, highlighting the persistence of a favourable clinical outcome despite the initial non-ideal acetabular reconstruction, exploring the factors underlying this successful long-term result.

## Case report

**Figure 1:**
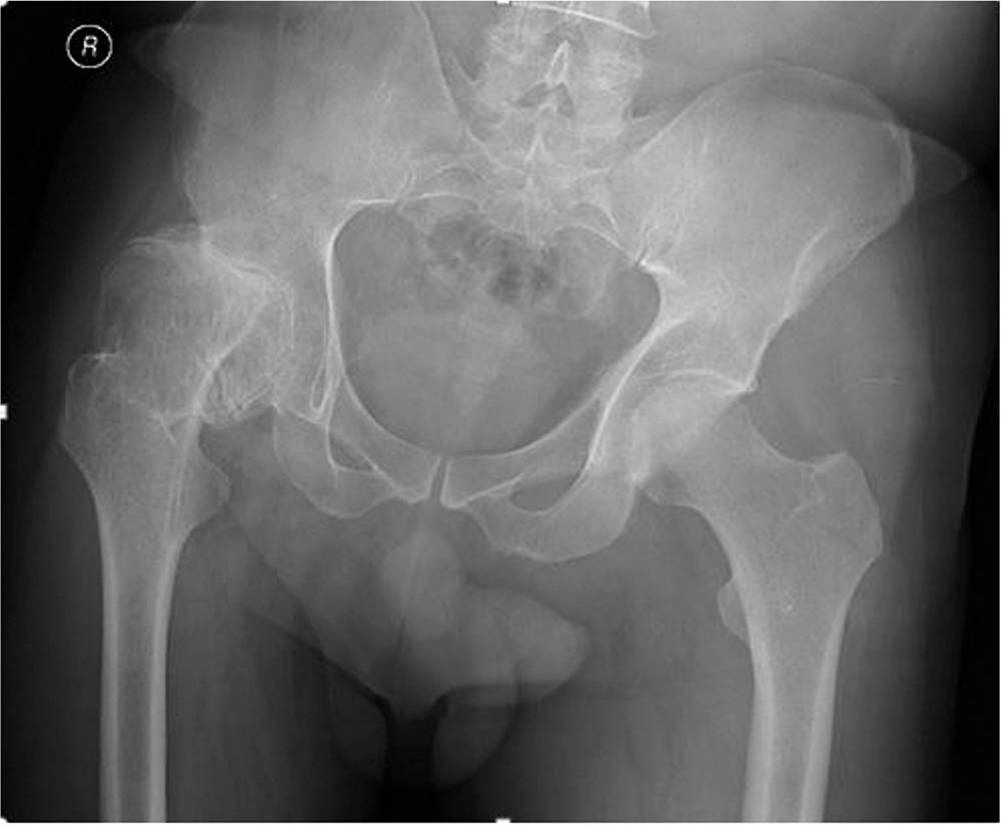
Preoperative anteroposterior radiograph of the pelvis demonstrating advanced right hip osteoarthritis (Tönnis 3), acetabular dysplasia (Crowe I), valgus hip, and pelvic obliquity.

In 2010, a 59-year-old male patient (BMI 26 kg/m^2^) presented to our department with severe right groin pain, functional impairment, a 30 mm limb-length discrepancy and Trendelenburg-type gait. The pelvic radiographs revealed right acetabular dysplasia (Crowe Grade I and Tönnis Grade 3), valgus hip, and pelvic obliquity (Fig. 1). A total hip prosthesis was performed via a standard posterior approach. A Müller roof reinforcement ring was implanted with a cemented polyethylene cup, and the femur was reconstructed using a cementless Wagner cone prosthesis stem.

Intraoperatively, an extensive superolateral portion of the metal ring remained uncovered by host bone, potentially compromising mechanical stability. The excised femoral head was morselised using a rongeur, and the autograft was impacted to fill the space between the ring and the native acetabular margin. The conical stem was implanted with adequate femoral anteversion.

**Figure 2:**
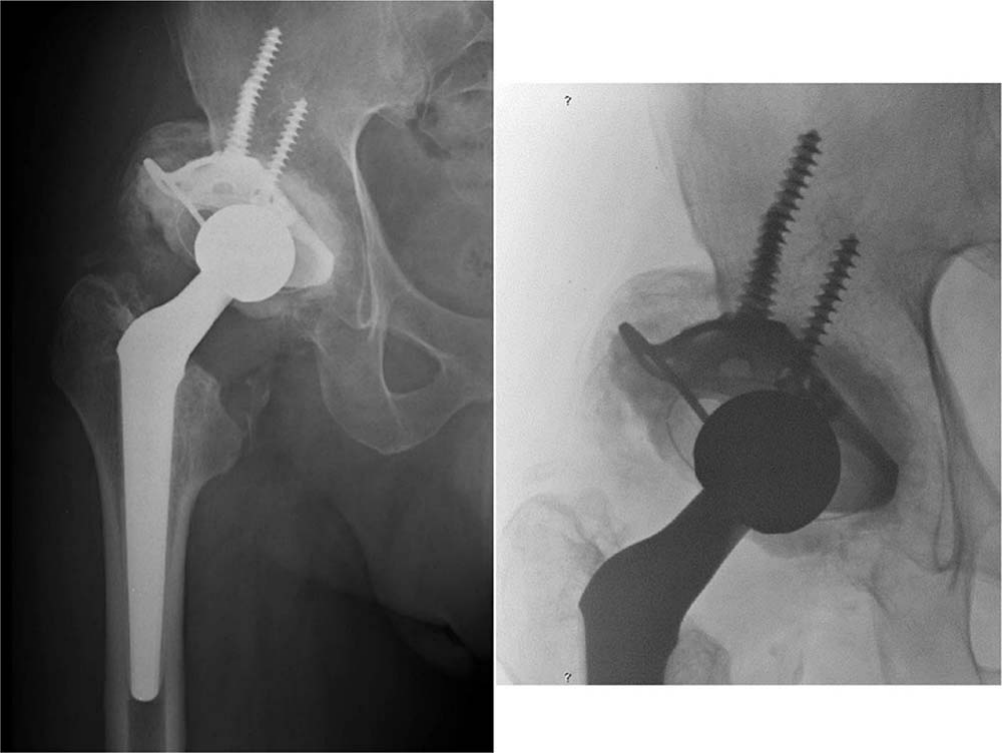
Postoperative THA radiograph at 1-year follow-up. A significant superolateral portion of the roof reinforcement ring remains uncovered by the native host bone. Evidence of the remodelling process is visible in the impacted morselised bone autograft, situated between the metal ring rim and the superior margin of the dysplastic acetabulum.

**Figure 3:**
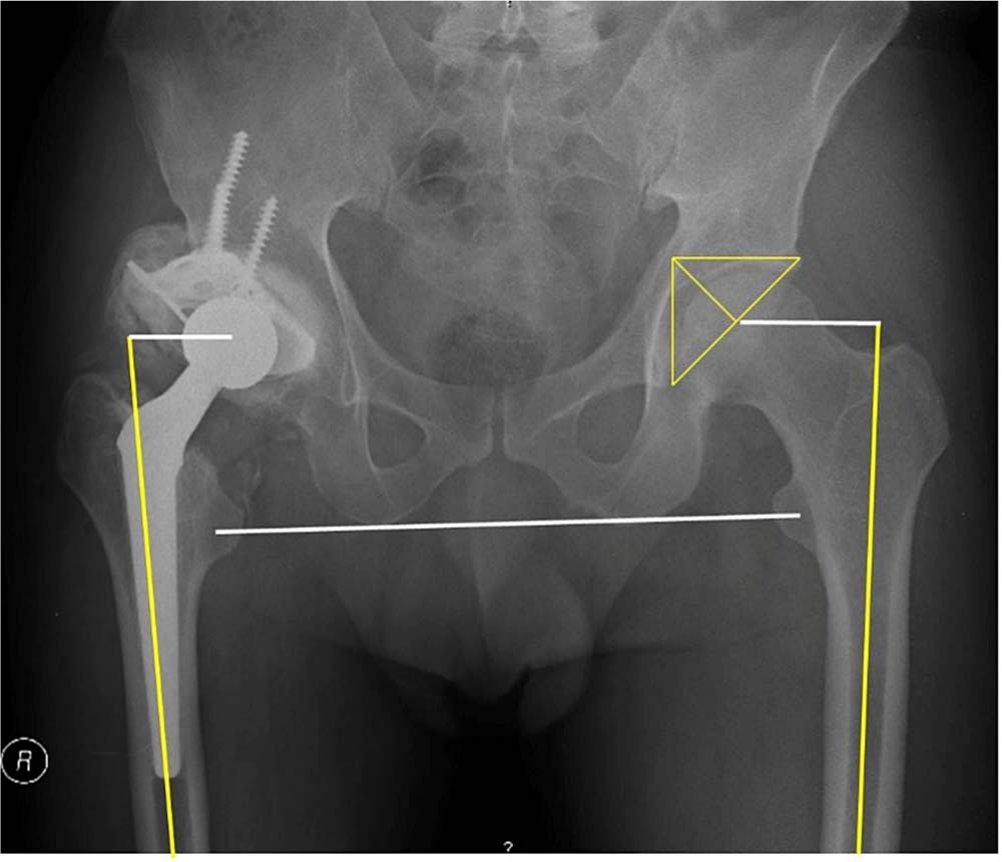
Postoperative THA radiograph at 5-year follow-up demonstrating stable implant fixation, absence of bone graft resorption, and acceptable restoration of the hip’s rotation centre and offsets. Correct inclination of the cemented polyethylene cup and near-equalisation of limb length are maintained.

The postoperative course was uneventful. One year postoperatively, the patient was asymptomatic with no limp and near-equalised limb lengths. Radiographs showed signs of bone autograft remodelling (Fig. 2). At 5-year follow-up, radiographs demonstrated stable fixation of the components, no subsidence, and no radiolucent lines. Heterotopic ossification was classified as Brooker stage I, and the autograft showed no resorption (Fig. 3).

## Results

**Figure 4:**
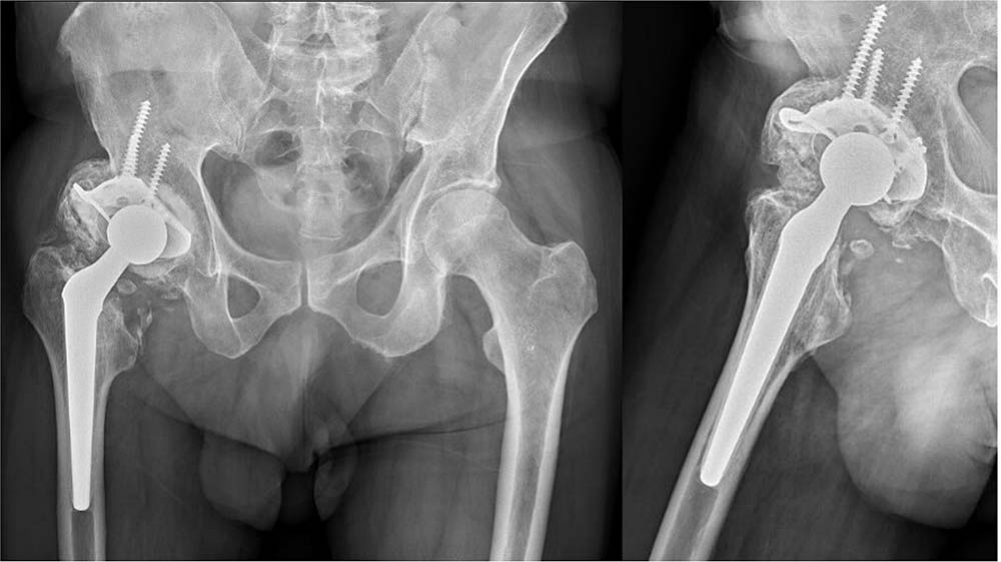
Postoperative THA radiographs at 16-year follow-up (2026) confirming complete autograft incorporation and well-fixed components. Despite moderate acetabular wear and mature heterotopic bone, no signs of implant loosening or instability are present.

At 16-year follow-up, the patient maintained excellent function (flexion 85°, extension 0°). Anteroposterior and lateral radiographs confirmed autograft incorporation and no signs of instability or loosening, despite moderate acetabular wear and mature heterotopic bone (Fig. 4). No cross-sectional imaging was performed, as the patient remained asymptomatic and routine institutional follow-up was based on plain radiographic assessment.

## Discussion

Bone deficiency on the superolateral border of the acetabulum is common in patients with DDH. During THA, this defect often represents the primary challenge in obtaining initial stability of the acetabular component [3, 4]. To address this, several methods have been developed, including superior displacement of the anatomical rotation centre of the hip, medialisation, impaction particulate bone grafting, structural bone grafting, reinforcement rings, cementless cups, and metal acetabular augments [4, 5].

When the acetabulum is shallow, reconstruction with roof reinforcement rings may be indicated. Although initially designed for revision cases, these rings are have also been used for primary THA with acetabular deficiencies [7–9]. Ideally, the acetabular component should be adequately medialised, the reinforcement ring should be sufficiently supported and covered by host bone and medialised to ensure mechanical stability. In our case, a polyethylene cup cemented into a Müller roof reinforcement ring was used. However, due to suboptimal acetabular reaming and insufficient medialisation, a significant superolateral portion of the ring remained uncovered, potentially compromising initial stability. Therefore, this reconstruction should not be interpreted as an ideal technical solution, but rather as a non-ideal acetabular reconstruction with an unexpectedly favourable long-term outcome. Intraoperative fluoroscopy was not utilised. With current techniques, such challenges may be reduced through improved preoperative planning, intraoperative imaging, or robotic-assisted navigation, allowing more accurate medialisation and acetabular component positioning.

To improve stability, morselised autogenous femoral-head graft was impacted to fill the uncovered void. Unlike structural grafting, impaction bone grafting has shown favourable mid-to-long-term effects in treating bone defects during THA for DDH [10]. We prefer morselising the graft using a rongeur rather than a bone mill, as a chip size between 5 mm and 8 mm is optimal for incorporation. Prior to grafting, the host bone surface must be prepared by drilling and roughening [11, 12]. While impaction grafting does not provide immediate mechanical stability, its biological incorporation ensures essential secondary stabilisation for long-term implant survival.

Contrary to acetabular side, the reconstruction of the femur was similar to that of conventional cases. We utilised a cementless conical femoral stem with a rounded cross-section. At 16-year follow-up, stable fixation and an excellent clinical result were maintained. The Wagner cone prosthesis is highly recommended for patients with challenging proximal femoral anatomy, small femoral diameter or poor metaphyseal bone quality [13, 14].

At 16 years postoperatively, the patient (now with 75-years-old) remains asymptomatic, with no limp and high satisfaction. Correcting limb-length discrepancy remains a technical priority to prevent complications like lower back pain and patient dissatisfaction, a common driver of orthopaedic litigation.

Cross-sectional imaging would have provided a more detailed assessment of heterotopic ossification and acetabular component version. However, CT was not performed at the 16-year follow-up because the patient was asymptomatic, clinically stable and showed no radiographic signs of loosening or instability. To improve the radiographic assessment, a lateral view was included in the final follow-up imaging, allowing additional evaluation of heterotopic ossification and component orientation.

## Conclusion

We attribute the success of the prosthesis stability and functional longevity to: successful bone autograft incorporation; correct inclination angle of the roof reinforcement ring and well-fixation with five screws; appropriate inclination angle of the cemented acetabular cup; acceptable medialization of the hip’s centre of rotation; favourable patient BMI, and a less physically demanding profession as a watchmaker. Nevertheless, further long-term follow-up is necessary to fully determine the mechanical behavior of the implants.
